# Saunders’ Question Compends

**Published:** 1902-07

**Authors:** 


SAUNDERS' QUESTION COMPENDS.—Essentials of Physiology. Prepared especially for students of medicine and arranged with questions following each chapter. By Sidney P. Budgett, M. D., professor of physiology, medical department of Washington University, St. Louis. 16mo volume of 233 pages, finely illustrated with many full-page half-tones. Philadelphia and London: W. B. Saunders & Co., 1901. Cloth, $1 net.

This handy little volume is conveniently arranged and tersely written. It is well adapted to meet the needs of the hard pressed undergraduate and all others who are anxious to gain a knowledge of the essentials of physiology at the sacrifice of the least possible amount of time and effort.

At the end of each chapter is a list of questions which review thoroughly the preceding pages, thus forming an excellent self-quiz.

For so small a work the illustrations are unusually good. The half-tones illustrating the various parts of the nervous system are as good as can be found in any text-book. R.

